# *Anaerococcus ihuae* sp. nov. and *Mediannikoviicoccus vaginalis* gen. nov., sp. nov.*,* two new bacteria isolated from human vaginal samples

**DOI:** 10.1007/s00203-022-03082-7

**Published:** 2022-07-20

**Authors:** Claudia Ly, Linda Abou Chacra, Eva Birsal, Gabriel Haddad, Cheikh Ibrahima Lo, Nicholas Amstrong, Stéphane Alibar, Blandine Courbière, Florence Bretelle, Florence Fenollar

**Affiliations:** 1 Aix Marseille Univ, IRD, AP-HM, SSA, VITROME, Marseille, France; 2 Institut Hospitalo-Universitaire Méditerranée-Infection, 19-21 Boulevard Jean Moulin, 13005 Marseille, France; 3 Aix-Marseille Univ, IRD, AP-HM, MEPHI, Marseille, France; 4 Department of Gynecology and Obstetrics, Gynépole, La Conception, AP-HM, Marseille, France

**Keywords:** *Anaerococcus ihuae* sp. nov., *Mediannikoviicoccus vaginalis* gen. nov., sp. nov, Anaerobic, Vaginal microbiota, Taxonomy

## Abstract

Strains Marseille-Q5893 (= CSUR Q5893 = CECT 30496) and Marseille-Q5883 (= CSUR Q5883 = CECT 30497) were isolated from vaginal samples using the culturomics approach. The 16S rRNA gene sequences of each strain were sequenced and then compared by BLASTn to the NCBI database. Strains Marseille-Q5893 and Marseille-Q5883 were most closely related to *Anaerococcus obesiensis* and *Finegoldia magna*, with identities of 98.5% and 90.0%, respectively. Strain Marseille-Q5893 is strictly anaerobic, while strain Marseille-Q5883 is facultative anaerobic. Both strains are Gram-positive, coccus-shaped, oxidase- and catalase-negative. The most abundant fatty acid for both strains is hexadecanoic acid, followed by 9-octadecenoic acid and tetradecanoic acid. Strain Marseille-Q5893 has a genome size of 1,831,271 bp with a G+C content of 29.4 mol%, whereas strain Marseille-Q5883 has a genome of 1,997,945 bp with a 33.6 mol% G+C content. The genomic comparison of closely related species with strains Marseille-Q5893 and Marseille-Q5883 showed that all digital DNA–DNA hybridization (dDDH) and orthologous average nucleotide identity (OrthoANI) values were lower than the published species thresholds (70% and 95–96%, respectively). Based on these data, we conclude that strain Marseille-Q5893 belongs to a new species in the family *Peptoniphilaceae* and strain Marseille-Q5883 belongs to a new genus in the family *Peptostreptococcaceae*. For these two new bacterial species, the names *Anaerococcus ihuae* sp. nov. and *Mediannikoviicoccus vaginalis* gen. nov., sp. nov., were proposed.

**Supplementary Information:**

The online version contains supplementary material available at 10.1007/s00203-022-03082-7.

## Introduction

The vaginal ecosystem is defined as all the microorganisms present in the vagina. It is characterized by the predominance of *Lactobacillus* spp., which represents approximately 90–95% of vaginal bacteria (Ravel et al. 2011; Abou Chacra and Fenollar 2021). Bacterial vaginosis represents a unique disturbance of this complex vaginal ecosystem, with the disappearance of *lactobacilli* and the proliferation of anaerobic bacteria, probably due to hormonal, behavioral, or environmental factors (Abou Chacra et al. 2022). This can have psychological (Kenyon et al. 2013), gynecological (Norenhag et al. 2020; Soper 2020) and obstetrical (Fox and Eichelberger 2015; Beckers and Sones 2020) consequences, in particular prematurity (Fettweis et al. 2019; Bayar et al. 2020). Indeed, several studies have shown that an imbalance of the vaginal flora leads to a proliferation of pathogenic microorganisms from the cervix to the choriodecidual space that leads to inflammation, with fragility of the fetal membranes and shortening of the cervix (Côté and Pasquier 2018).

Using the “culturomics” approach, a culture technique consisting in incubating samples under different culture conditions and subsequently identifying the isolated species by matrix-assisted desorption ionization–time of flight mass spectrometry (MALDI-TOF MS) (Lagier and Raoult 2016), we isolated a new member of the genus *Anaerococcus* that did not correspond to the other species of this genus and a new genus *Mediannikoviicoccus* from vaginal samples. These strains are referenced as Marseille-Q5893 and Marseille-Q5883, respectively.

Herein, we reported the description of these two strains in pure culture, Marseille-Q5893 and Marseille-Q5883, according to the new polyphasic approach named taxonogenomic, which combines annotated whole genome and proteomic information obtained from MALDI-TOF MS spectra and phenotypic characteristics.

## Materials and methods

### Ethical approval and isolation of strains

Strain Marseille-Q5893 was isolated from a vaginal sample of a 30-year-old non-pregnant woman, whereas strain Marseille-Q5883 was isolated from a vaginal sample from a 23-year-old pregnant woman. These two women had no bacterial vaginosis or sexually transmitted infection at the time of the consultation. The study was approved by the local ethics committee of the Institut Hospitalo-Universitaire Méditerranée Infection (Marseille, France) under agreement number 2021-016. The patients provided signed informed consent.

The culture of strains Marseille-Q5893 and Marseille-Q5883 was achieved after pre-incubation of the vaginal samples at 37 °C in anaerobic blood culture vials (Becton Dickinson, Le Pont-de-Claix, France) supplemented with 40 mL of Difco Marine Broth (Becton Dickinson) for 7 days and 21 days, respectively. Then, isolated colonies were obtained by subculture on 5% sheep blood-enriched Columbia agar (bioMérieux, Marcy l’Etoile, France) at 37 °C in an anaerobic atmosphere using AnaeroGen (bioMérieux) after 48 h.

### Identification of strains and phylogenetic analysis

Identification of strains Marseille-Q5893 and Marseille-Q5883 was carried out using a Microflex LT MALDI-TOF mass spectrometer (Bruker Daltonics, Bremen, Germany) (Seng et al. 2013). The spectra of the two strains were imported into the MALDI Biotyper software (version 2.0, Bruker) and analyzed by standard pattern matching (with default parameter settings). Interpretation of the scores was performed as previously described (Hadjadj et al. 2016).

The 16S rRNA gene of strains Marseille-Q5893 and Marseille-Q5883 was sequenced using the fD1 and rP2 universal primers (Eurogentec, Angers, France) as previously described (Drancourt et al. 2000), using an ABI Prism 3130xl Genetic Analyzer capillary sequencer (Thermo Fisher, Saint-Aubin, France). If the 16S rRNA sequence similarity with closely related species was between 95 and 98.65%, the strain was suggested as belonging to a new species (Stackebrandt and Goebel 1994; Kim et al. 2014). If the similarity was below 95%, the strain would be considered a new genus (Tindall et al. 2010; Rossi-Tamisier et al. 2015).

Using the MEGA-X (Kumar et al. 2018), the resulting 16S rRNA gene sequences were aligned and a phylogenetic tree was obtained with 1000 bootstrap replicates, based on the maximum likelihood (ML) and the Kimura 2-parameter methods (Kimura 1980).

### Morphological and phenotypic characterization

The morphology of the both species was observed via a SU5000 scanning electron microscope (SEM; Hitachi High-Technologies, Tokyo, Japan) as previously described (Zgheib et al. 2021). The phenotypic characteristics of both strains such as Gram staining, motility, oxidase, and catalase activities were determined after incubation on 5% sheep blood-enriched Columbia agar (bioMérieux) at 37 °C in anaerobic atmosphere for 48 h. Gram staining of cells was carried out using a Color Gram 2 kit (bioMérieux). Catalase activity was determined by observing bubble production after the application of 3% (v/v) hydrogen peroxide solution. Oxidase activity was evaluated via the oxidation of 1% (w/v) p amino-dimethylaniline oxalate. The spore formation test was also performed on strains after a shock for 20 min at 80 °C.

The optimal growth conditions of both strains were determined by culturing each strain under different atmospheres, temperatures, pH, and salinity parameters. The strains were cultivated and incubated under aerobic, anaerobic (GENbag anaer, bioMérieux), and micro-aerophilic (GENbag microaer, bioMérieux) atmospheres on 5% sheep blood-enriched Columbia agar (bioMérieux) and at the following temperatures: ambient, 28, 37, 42, and 56 °C. The pH conditions used were 5.5, 6, 6.5, 7, 7.5, 8, and 8.5. The salinity conditions employed were 0%, 5%, 7.5%, 10%, 15%, and 20%.

The biochemical properties were evaluated using rapid API ZYM, API 20A, and API 50CH strips (bioMérieux) according to the manufacturer’s instructions. Antibiotic susceptibility was determined using E-test gradient strips (bioMérieux) according to the EUCAST recommendations (Matuschek et al. 2014). Finally, cellular fatty acid methyl ester (FAME) analysis was performed by chromatography/mass spectrometry GC/MS as previously reported (Sasser 2006; Dione et al. 2016).

### Genome extraction, sequencing, annotation, and comparison

To extract genomic DNA, the strains Marseille-Q5893 and Marseille-Q5883 were mechanically treated with acid-washed glass beads (G4649-500g, Sigma-Aldrich, Saint-Quentin-Fallavier, France) by a FastPrep BIO 101 instrument (Qbiogene, Strasbourg, France) at maximum speed (6.5 m/s) for 90 s, followed by 2-h lysozyme incubation at 37 °C. Then, the DNA was extracted using the EZ1 BioRobot and the EZ1 DNA Tissue kit (Qiagen, Hilden, Germany).

The sequencing of strains Marseille-Q5893 and Marseille-Q5883 was performed using a MiSeq sequencer (Illumina Inc., San Diego, CA, USA) via the Nextera Mate Pair sample prep kit and Nextera XT Paired End (Illumina) as previously reported (Anani et al. 2019). SPAdes 3.13.1 software was used with default parameters to assemble the reads (Bankevich et al. 2012). Scaffolds with a nucleotide number < 800 bp and scaffolds with a depth value < 25% of the mean depths were removed. The resulting genome for each strain and the genomes of closely related species were annotated with Prokka 1.14.5 as previously described (Seemann 2014; Zgheib et al. 2020). The genome as well as 16S rRNA sequences extracted from the genomes were compared to closely related species (Table 2).

In addition, digital DNA–DNA hybridization (dDDH) was applied using the Genome-to-Genome Distance Calculator (GGDC) 2.1 web server (http://ggdc.dsmz.de/distcalc2.php) to estimate the overall similarity among the compared genomes (Auch et al. 2010; Meier-Kolthoff et al. 2013). Average nucleotide identity analysis was also estimated via OrthoANI 1.2 (Lee et al. 2016). The species thresholds for dDDH and OrthoANI are 70% and 95–96%, respectively (Meier-Kolthoff et al. 2013; Kim et al. 2014).

Furthermore, the CRISPRCasFinder program was used to easily detect clustered regularly interspaced short palindromic repeats (CRISPRs) and *cas* (CRISPR-associated) genes in both strains (Grissa et al. 2007). The PathogenFinder 1.1 program was also applied to identify gene families that correlate with pathogenicity (Cosentino et al. 2013).

## Results

### Strain identification and phylogenetic analysis

**Fig. 1:**
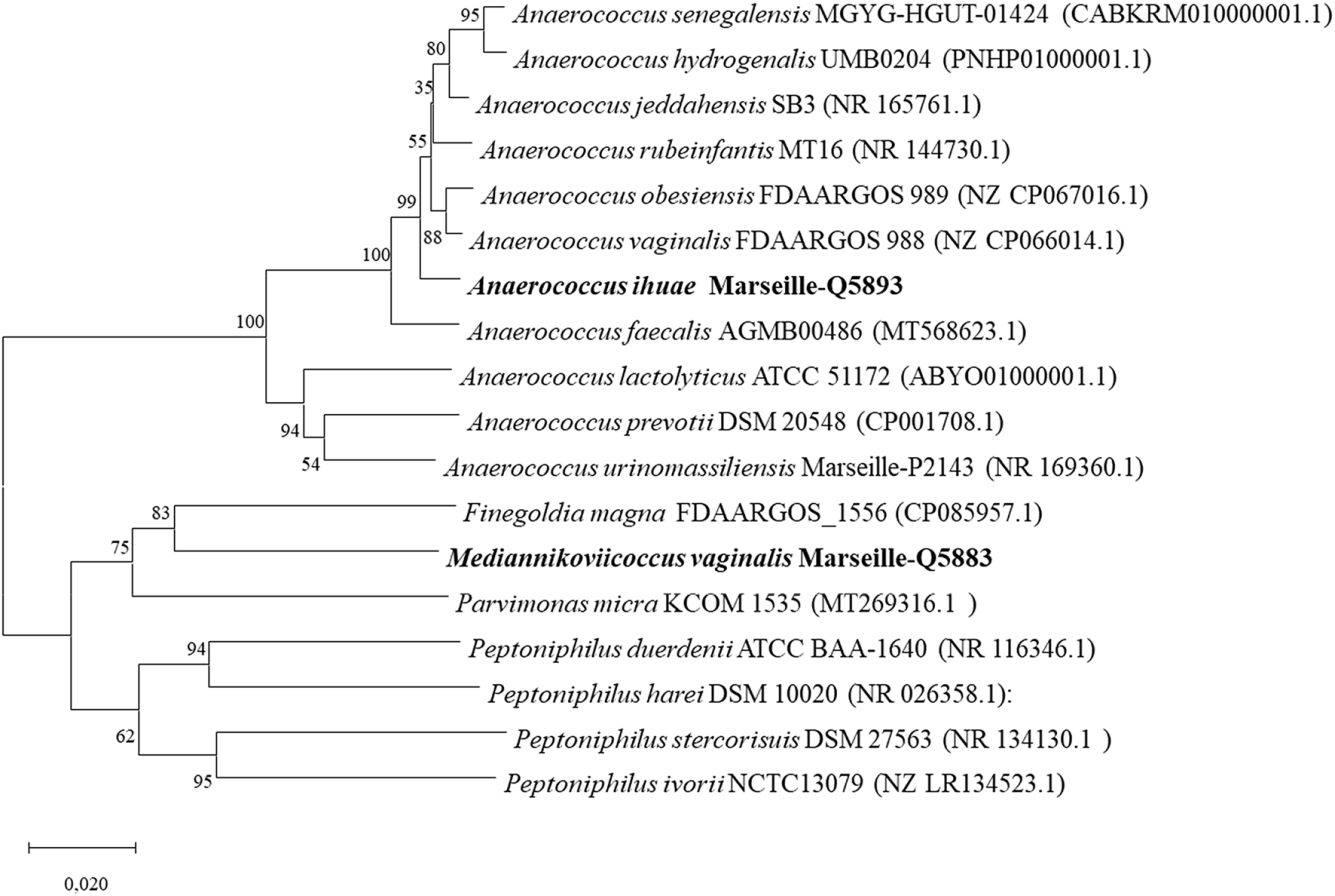
16S rRNA-based phylogenetic tree inferred from the comparison of 16S rRNA gene sequences of strains *Anaerococcus ihuae Marseille-*Q5893 and *Mediannikoviicoccus vaginalis Marseille-*Q5883 (bold) and closely related species. Accession numbers of the genomes where 16S rRNA gene sequences were extracted are indicated in parentheses. The sequences were aligned using MUSCLE. The tree was generated with the MEGA-X software using the ML method and Kimura 2-parameter model (16,17). The scale bar indicates 10% sequence divergence. Numbers at the nodes indicate bootstrap value

Strains Marseille-Q5893 and Marseille-Q5883 could not be identified by our MALDI-TOF MS instrument, as the score was lower than 1.8, suggesting that the corresponding species was not referenced in the database and could be an unknown species (Fig. S1). Additionally, strain Marseille-Q5893 revealed a 98.5% 16S rRNA sequence similarity with *Anaerococcus obesiensis* strain FDAARGOS_989 (CP067016.1), the phylogenetically closest species with standing in the nomenclature. As this value was below the 98.65% threshold for defining a new bacterial species, strain Marseille-Q5893 was considered as a representative of a putatively new species within the family *Peptoniphilaceae* in the phylum *Firmicutes.* Similarly, strain Marseille-Q5883 revealed a 90.0% 16S rRNA sequence similarity with *Finegoldia magna* strain FDAARGOS_1556 (CP085957.1), the phylogenetically closest species with standing in the nomenclature. As this value was below the 95% threshold for defining a new bacterial genus, strain Marseille-Q5883 was considered as a representative of putatively new genus within the family *Peptostreptococcaceae* in the phylum *Firmicutes.* The phylogenetic trees highlighting the position of each of the two strains relative to other closely related species with a validly published name are shown in Fig. 1.

### Phenotypic characterization

The optimal growth of strains Marseille-Q5893 and Marseille-Q5883 was obtained after 2 days of culture at 37 °C under anaerobic conditions (anaeroGEN, Oxoid Ltd, Dardilly, France). In these culture conditions, strains Marseille-Q5893 formed circular, white, and opaque colonies with a diameter of 2–2.5 mm. Colonies from strain Marseille-Q5883 appear circular, white, and translucent, with a diameter of 1.5–2 mm. With the strain Marseille-Q5883, growth occurs also under a micro-aerophilic atmosphere but not for strain Marseille-Q5893.

**Fig. 2:**
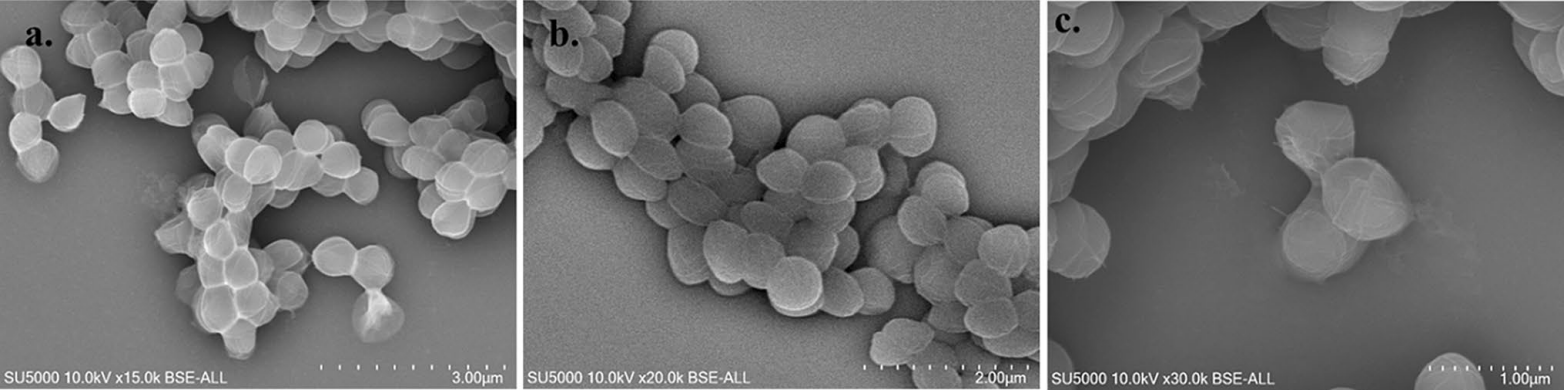
Scanning electron microscopy of *Mediannikoviicoccus vaginalis* gen. nov., sp. nov., strain Marseille-Q5883 (**a**, **b**, and **c**)

Bacterial cells observed by SEM are nearly 0.75 ± 0.07 μm in diameter and disposed in clusters for strain Marseille-Q5893 (Fig. S2a, S2b, S2c), and nearly 0.62 ± 0.10 μm in diameter and occur in pairs or short chains for strain Marseille-Q5883 (Fig. 2a–c).

The characteristics of strain Marseille-Q5893 and strain Marseille-Q5883 are summarized in supplementary Table S1.

For strain Marseille-Q5893, using API ZYM strips, positive results were obtained for leucine arylamidase and acid phosphatase. Using API 50CH strips, acid is not produced from glucose or other sugars. All remaining reactions were negative with API 20A strips (Table 1, Table S2). The major fatty acids were comparable to previously described *Anaerococcus* species: hexadecanoic acid (61%), 9-octadecenoic acid (20%), and tetradecanoic acid (6%). Several fatty acids with shorter chains were also detected with lower amounts (C8–C10–C12). A few branched structures were also listed at lower abundances (Table S3). The minimum inhibitory concentration was 0.032 μg/L for penicillin G, 0.032 μg/L for amoxicillin, 0.25 μg/L for cefotaxime, 12 μg/L for ceftazidime, 0.023 μg/L for imipenem, 0.125 μg/L for oxacillin, 0.125 μg/L for daptomycin, 1.5 μg/L for doxycycline, 0.003 μg/L for rifampicin, 0.023 μg/L for teicoplanin, and 4 μg/L for vancomycin. In addition, strain Marseille-Q5893 was resistant to amikacin, ciprofloxacin, clindamycin, sulfamethoxazole-trimethoprim, and tobramycin.

**Table 1:** Comparison of *Anaerococcus ihuae* sp. nov., strain Marseille-Q5893 and *Mediannikoviicoccus vaginalis* gen. nov., sp. nov., strain Marseille-Q5883 with its phylogenetically closest species with a validly published name

Properties	1	2	3	4	5	6	7	8	9
Cell size (μm)	0.75	0.71	0.9	0.85–1.2	0.78	0.87	0.7	0.3–0.7	0.8–1.6
O_2_ requirement	Anaerobic	Anaerobic	Anaerobic	Anaerobic	Anaerobic	Anaerobic	Anaerobic	Anaerobic	Anaerobic
Gram stain	+	+	+	+	+	+	+	+	+
Mobility	−	−	−	−	−	−	−	−	−
Catalase	−	+	+	−	−	−	−	Variable	−
Production of									
Acid phosphatase	+	−	+	+	−	−	−	−	−
Alkaline phosphatase	−	−	+	+	−	−	−	+	d
Arginine dihydrolase	−	+	−	−	−	−	−	−	d
Glycine arylamidase	−	w	−	−	−	−	−	+	+
Histidine arylamidase	−	+	−	−	−	−	−	+	−/w
Naphthol-AS-BI-phosphohydrolase	−	−	+	+	+	−	−	+	+
Pyroglutamic acid arylamidase	−	−	−	−	−	−	+	−	+
Valine arylamidase	−	−	+	+	−	−	−	+	−
α-Glucosidase	−	−	−	+	−	−	−	−	−
ß-Glucosidase	−	−	−	+	−	−	−	−	−
ß-Glucuronidase	−	−	−	+	−	−	−	−	−
Utilization of									
d-Glucose	−	−	+	+	−	−	−	−/w	−/w
d-Sucrose	−	−	+	-	−	−	−	−	−
d-Mannose	−	+	+	+	−	−	−	−	−
G+C content (mol%)	29.4	29.6	29.5	29.7	33.6	30.1	33.4	27–29	33
Habitat	Human vagina	Human vagina	Human gut	Human gut	Human vagina	Human gut	Human vagina	Human gut	Human gut

Strains: **1**, *Anaerococcus ihuae* Marseille-Q5893; **2**, *Anaerococcus vaginalis* PH9 (Hugon et al. 2012); **3**, *Anaerococcus rubiinfantis* mt16 (Alou et al. 2016); **4**, *Anaerococcus jeddahensis* SB3 (Dione et al. 2018)*;* **5**, ***Mediannikoviicoccus**** vaginalis* Marseille-Q5883; **6**, *Peptoniphilus obesi* ph1 (Mishra et al. 2013); **7**, *Peptoniphilus duerdenii* WAL 18,896 (Ulger-Toprak et al. 2012); **8**, *Parvimonas micra* ATCC 33270 (Murdoch and Shah 1999); **9**, *Finegoldia magna* ATCC 15794 (Murdoch and Shah 1999); + , positive; − negative; w, weakly positive; d, different reactions

For strain Marseille-Q5883, using API ZYM strips, positive results were observed for leucine arylamidase and naphthol-AS-BI-phosphohydrolase. Using API 50 CH strips, acid was not produced from glucose or other sugars. All remaining reactions were still negative with API 20A strips (Table 1, Table S2). The most abundant fatty acid by far was hexadecanoic acid (62%), followed 9-octadecenoic acid (15%), and tetradecanoic acid (8%). Minor amounts of unsaturated, branched, and saturated fatty acids were also described (Table S3). The minimum inhibitory concentration was 0.38 μg/L for penicillin G, 0.032 μg/L for amoxicillin, 0.094 μg/L for imipenem, 4 μg/L for oxacillin, 0.38 μg/L for ciprofloxacin, 0.064 μg/L for clindamycin, 0.064 μg/L for daptomycin, 1.5 μg/L for doxycycline, 0.002 μg/L for rifampicin, 0.032 μg/L for tobramycin, 0.094 μg/L for teicoplanin, and 4 μg/L for vancomycin. In addition, strain Marseille-Q5883 was resistant to amikacin, cefotaxime, ceftazidime and sulfamethoxazole–trimethoprim.

### Genomic analysis

**Fig. 3:**
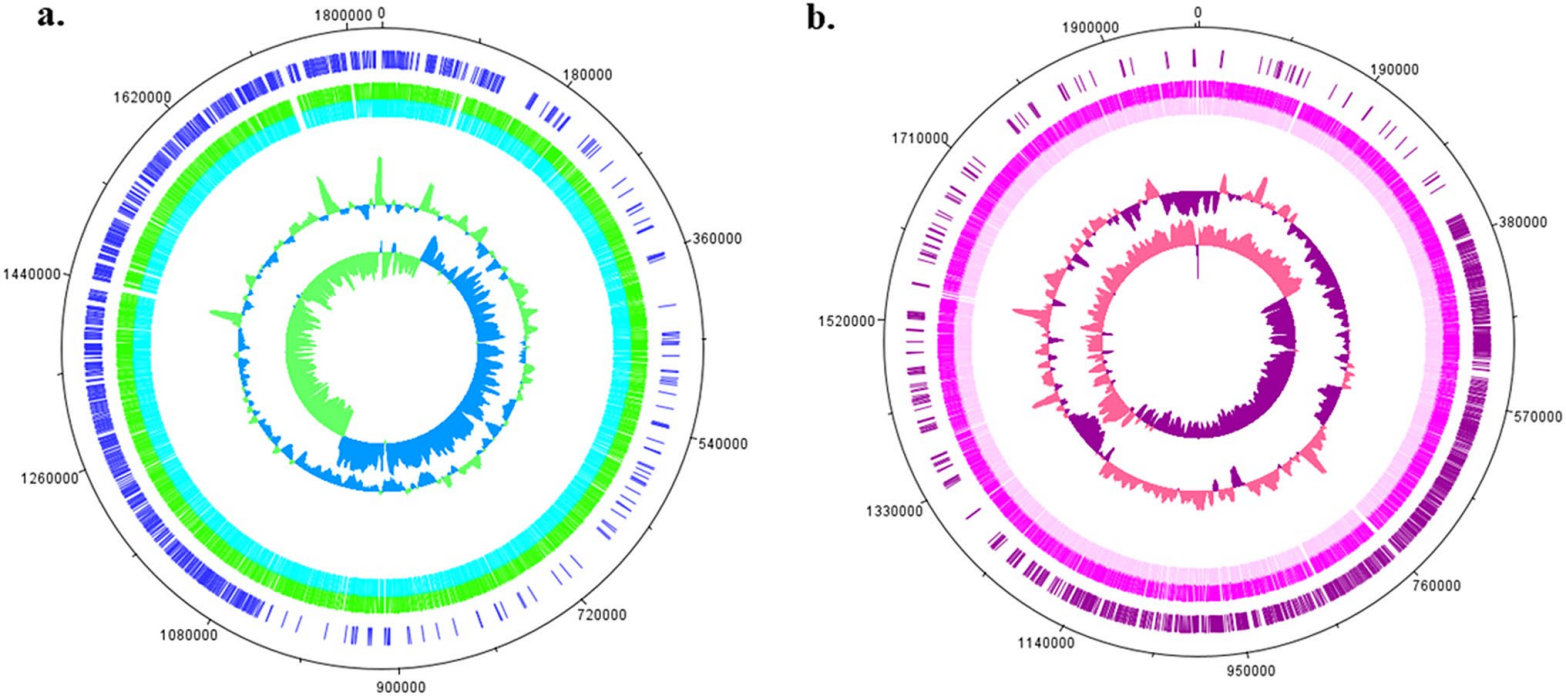
Graphical circular map of genomes of **a** *Anaerococcus ihuae* sp. nov., strain Marseille-Q5893 and **b** *Mediannikoviicoccus vaginalis* gen. nov., sp. nov., strain Marseille-Q5883

The number of reads for *Anaerococcus ihuae* strain Marseille-Q5893 was 2,322,216 with a coverage of 50 ×. The genome length was 1,831,271 bp, assembled into 5 contigs, with a G+C content of 29.4 mol% (Fig. 3a). Strain Marseille-Q5893 has 1748 predicted genes, including 1687 protein-coding genes. Strain Marseille-Q5893 also had 61 RNA-coding genes, including 9 rRNA, 51 tRNA, and 1 tmRNA.

The number of reads for *Mediannikoviicoccus vaginalis* strain Marseille-Q5883 was 1,872,862 with a coverage of 50 ×. The genome length was 1,831,271 bp, assembled into 5 contigs, with a G+C content of 29.4 mol% (Fig. 3a). Strain Marseille-Q5893 has 1748 predicted genes, including 1687 protein-coding genes. Strain Marseille-Q5893 also had 61 RNA-coding genes, including 9 rRNA, 51 tRNA, and 1 tmRNA.

The genome length of *Mediannikoviicoccus vaginalis* strain Marseille-Q5883 was 1,997,945 bp, assembled into 5 contigs, with a G+C content of 33.6 mol% (Fig. 3b). Strain Marseille-Q5883 has 1852 predicted genes including 1795 protein-coding genes. Strain Marseille-Q5883 also had 57 RNA-coding genes, including 12 rRNA, 44 tRNA, and 1 tmRNA. In addition, the genomic characteristics of these new strains are statistically compared with other related species (Table 2).

**Table 2:** Summary of genome properties for compared strains

Strains	Accession	Size (bp)	Coding region (bp)	G+C (%)	Total genes	Protein-coding genes	rRNAs	tRNAs
1	CAKMRU010000001.1	1,831,371	1,651,651	29.42	1748	1687	9	51
2	CAKMRI010000001.1	1,997,945	1,812,708	33.62	1852	1795	12	44
3	ABYO01000001.1	2,177,754	1,887,479	34.94	2066	2018	3	44
4	NZ_CP067016.1	1,893,092	1,680,889	28.98	2003	1944	9	49
5	NZ_CP066014.1	1,893,964	1,722,408	28.96	1778	1719	9	49
6	NZ_CP085957.1	1,738,818	1,602,234	32.4	1695	1634	12	48
7	NZ_CP009761.1	1,627,009	1,447,647	28.61	1537	1484	11	41
8	CABKRE010000001.1	1,773,998	1,580,910	30.15	1697	1668	2	26
9	JAGGLJ010000001.1	1,795,400	1,623,528	27.87	1774	1714	4	55
10	CAHE01000001.1	1,726,703	1,497,432	30.68	1890	1867	3	19

Strains: **1**, *Anaerococcus ihuae* sp. nov., Marseille-Q5893; **2**, *Mediannikoviicoccus vaginalis* sp. nov., Marseille-Q5883; **3**, *Anaerococcus lactolyticus* ATCC 51172*;* **4**, *Anaerococcus obesiensis* FDAARGOS_989; **5**, *Anaerococcus vaginalis* FDAARGOS_988; **6**, *Finegoldia magna* FDAARGOS_1556; **7**, *Parvimonas micra* KCOM 1535; **8**, *Peptoniphilus obesi* MGYG-HGUT-01414; **9**, *Peptoniphilus stercorisuis* DSM 27563; **10**, *Peptoniphilus timonensis* JC401

The most elevated value of dDDH for strain Marseille-Q5893 against the accessible genomes for type strains of species, with standing in the nomenclature, is 53% with *Anaerococcus rubeinfantis* (Table 3). For strain Marseille-Q5883, the most elevated value of dDDH is 54.9% with *Peptoniphilus obesi* (Table 3). These estimations are less than 70% of the cutoff utilized for delineating prokaryotic species, consequently affirming that these strains represent two new species, distinct from these other bacterial strains.

**Table 3:** dDDH values and OrthoANI values calculated by OAT software of strain Marseille-Q5893 (*Anaerococcus ihuae* sp. nov.) and strain Marseille-Q5883 (*Mediannikoviicoccus vaginalis* gen. nov., sp. nov.) with other closely related species with standing in nomenclature

Query strain	Subject strain	dDDH (%)	OrthoANI (%)	G+C content difference (%)
Marseille-Q5893	*Anaerococcus rubeinfantis*	53	93.47	0.03
	*Anaerococcus jeddahensis*	46.9	92.02	0.31
	*Anaerococcus vaginalis*	35.1	87.96	0.47
	*Anaerococcus obesiensis*	35	87.89	0.45
	*Anaerococcus hydrogenalis*	29.6	85.03	0.42
	*Anaerococcus senegalensis*	29.5	85.23	1.03
	*Anaerococcus lactolyticus*	25.6	70.79	5.51
	*Anaerococcus prevotii*	22.5	70.29	6.65
	*Parvimonas micra*	22.1	65.99	0.81
Marseille-Q5883	*Peptoniphilus obesi*	54.9	72.73	3.47
	*Finegoldia magna*	44.4	71.76	1.63
	*Anaerococcus vaginalis*	43.3	70.06	4.66
	*Anaerococcus obesiensis*	42.3	69.83	4.64
	*Peptoniphilus duerdenii*	37.6	72.09	0.62
	*Peptoniphilus timonensis*	35.4	67.82	2.94
	*Peptoniphilus ivorii*	28.1	63.49	19.6
	*Parvimonas micra*	24.4	67.17	5
	*Peptoniphilus stercorisuis*	23	65.48	5.75
	*Peptoniphilus asaccharolyticus*	3.7	62.66	6.82

OrthoANI values of strain Marseille-Q5893 ranged between 65.9 and 93.4%, confirming that this strain is different from the other tested bacterial strains (Table 3). In the aggregate, the above information supports strain Marseille-Q5893 as representing a novel species in the family *Peptoniphilaceae,* for which the name *Anaerococcus ihuae* sp. nov., is proposed. For strain Marseille-Q5883, OrthoANI values ranged from 63.49 to 72.73%, affirming again that this strain is different from the other tested bacterial strains (Table 3). Overall, the above information also supports that strain Marseille-Q5883 represents a new genus in the family *Peptostreptococcaceae*, for which the name *Mediannikoviicoccus vaginalis* gen. nov. is proposed.

Using the CRISPRCasFinder program, only the Marseille-Q5883 strain had a genomic structure corresponding to a CRISPR with a very high level of evidence. Indeed, a conserved region consisted of a 28-bp length sequence “GTTGTTCCTGCATGCAGGGGTGATCC” repeated 20 times in a 1,189-bp fragment and separated by 19 unique sequence of similar size (spacers) was detected. Moreover, the *cas* genes cluster (Type IE) was also evidenced.

Using the PathogenFinder 1.1 program, the strain Marseille-Q5893 was predicted as a human pathogen with a 0.7 probability, matched to 6 pathogenic protein families (4 hypothetical conserved protein and 1 putative transposon integrase from *Finegoldia magna* as well as 1 hypothetical conserved protein from *Streptococcus pyogenes*). Likewise, the strain Marseille-Q5883 was predicted as a human pathogen with a 0.8 probability, matched to 15 pathogenic protein families (1 putative peptidase, 1 putative chimeric erythrocyte-binding protein, 1 DNA topoisomerase III, 1 putative transposon integrase, 1 ABC transporter permease protein, 4 conserved hypothetical protein from *Finegoldia magna* as well as 1 Superfamily II DNA and RNA helicase, 1 site-specific recombinase and 3 conserved hypothetical protein from *Streptococcus pyogenes,* and 1 conserved hypothetical protein *Streptococcus pneumoniae* Taiwan19F-14).

## Conclusion

The 16S rRNA gene sequence identities between the two strains Marseille-Q5893 and Marseille-Q5883 and closely related species were less than 98.65% and 95%, respectively, the threshold delimiting a new bacterial species and a new bacterial genus, respectively. The dDDH values between the compared genomes were all below the recommended threshold of 70%. Therefore, according to phenotypic, phylogenetic, and genomic analyses, we state that the 2 new strains are new members belonging to Firmicutes group for which *Anaerococcus ihuae* sp. nov. and *Mediannikoviicoccus vaginalis* gen. nov. are proposed as their names.

### Description of *Anaerococcus ihuae* sp. nov.

*Anaerococcus ihuae* (i.hu’ae, N.L. gen. n. *ihuae,* based on the acronym IHU, the Institut Hospitalo-Universitaire Méditerranée-Infection in Marseille, France, where the type strain was isolated).

Cells are strictly anaerobic, Gram-positive, non-spore-forming, non-motile, and coccus-shaped. Bacterial cells are nearly 0.75 μm in diameter and disposed in clusters. Catalase and oxidase activities are negative. After 48 h incubation on Columbia agar supplemented with 5% sheep blood, colonies appear circular, white, and opaque with a diameter of 2–2.5 mm. Growth occurs only under an anaerobic atmosphere in a temperature range of 28–42 °C (optimum 37 °C), at pH 6–7.5 (optimum pH 7), and with 0–20% (w/v) NaCl (optimum 15–20%).

Using API ZYM strips, only leucine arylamidase and acid phosphatase enzyme activities are positive. Using API 50 CH strips, acid is not produced from glucose or other sugars. All remaining reactions were still negative with API 20E strips. The major fatty acids are hexadecanoic acid (61%), 9-octadecenoic acid (20%), and tetradecanoic acid (6%). The size of genome is 1.09 Mbp and its G+C content is 29.4 mol%.

The type strain Marseille-Q5893^T^ (= CSUR Q5893 = CECT 30,496) was isolated from a vaginal sample of a 30-year-old healthy woman at day 16 of the menstrual cycle.

The 16S rRNA and genome sequences are deposited in GenBank under accession numbers OM728648 and CAKMRU010000001, respectively.

### Description of *Mediannikoviicoccus* gen. nov.

*Mediannikoviicoccus* (Me.di.an.ni.ko.vi.i.coc'cus. N.L. masc. n. coccus (from Gr. masc. n. *kokkos*, a grain or seed), a coccus; N.L. masc. n. *Mediannikoviicoccus*, a coccus named in honor of the clinical microbiologist doctor Oleg Mediannikov).

Cells are facultative anaerobic, Gram-positive, non-spore-forming, non-motile, and coccus-shaped. Catalase and oxidase activities are negative. The major FAMEs (> 8%) are hexadecenoic, 9-octadecenoic acid and tetradecanoic acid. The genome size is 1.95 Mbp and its G+C content is 33.6 mol%. This genus is a member of the family *Peptoniphilaceae* with *Mediannikoviicoccus vaginalis* as the type species.

### Description of *Mediannikoviicoccus vaginalis* gen. nov., sp. nov.

*Mediannikoviicoccus vaginalis* (va.gi.na’lis. L. fem. n. vagina, sheath, vagina; N.L. masc. adj. *vaginalis*, pertaining to vagina).

Cells are facultative anaerobic, Gram-stain-positive, non-spore-forming, non-motile, and coccus-shaped. Bacterial cells are nearly 0.63 μm in diameter and occur in pairs or short chains. Catalase and oxidase activities are negative. After 48 h incubation on Columbia agar supplemented with 5% sheep blood, colonies are circular, white, and translucent with a diameter of 1.5–2 mm. Growth occurs under an anaerobic and micro-aerophilic atmosphere in a temperature range of 28–42 °C (optimum 37 °C), at pH 6–7.5 (optimum pH 7) and with 0–20% (w/v) NaCl (optimum 15–20%).

Using API ZYM strips, positive results were obtained for leucine arylamidase and naphthol-AS-BI-phosphohydrolase. Using API 50CH strips, acid is not produced from glucose or other sugars. The most abundant fatty acid by far was hexadecanoic acid (62%), followed 9-octadecenoic acid (15%), and tetradecanoic acid (8%). The genome size of strain Marseille-Q5883 is 1.95 Mbp and its G+C content is 33.6 mol%.

The type strain Marseille-Q5883^T^ (= CSUR Q5883 = DSM 30497) was isolated from a vaginal sample of a 23-year-old pregnant woman with threatened premature delivery with no identified etiology.

The 16S rRNA and genome sequences are deposited in GenBank under accession numbers OM728652 and CAKMRI010000001, respectively.

## Supplementary Information

Below is the link to the electronic supplementary material.

Supplementary file1 (DOCX 429 KB)
