# Supplementary material for: Anaerococcus ihuae sp. nov. and Mediannikoviicoccus vaginalis gen. nov., sp. nov., two new bacteria isolated from human vaginal samples
Source: Arch Microbiol. 2022 Jul 20;204(8):508. doi: 10.1007/s00203-022-03082-7 (PMC9300522; doi:10.1007/s00203-022-03082-7)
Supplement: Supplementary file 1 — Supplementary file1 (DOCX 429 KB) [file 203_2022_3082_MOESM1_ESM.docx]

**Supplementary data**

**Figures**

**
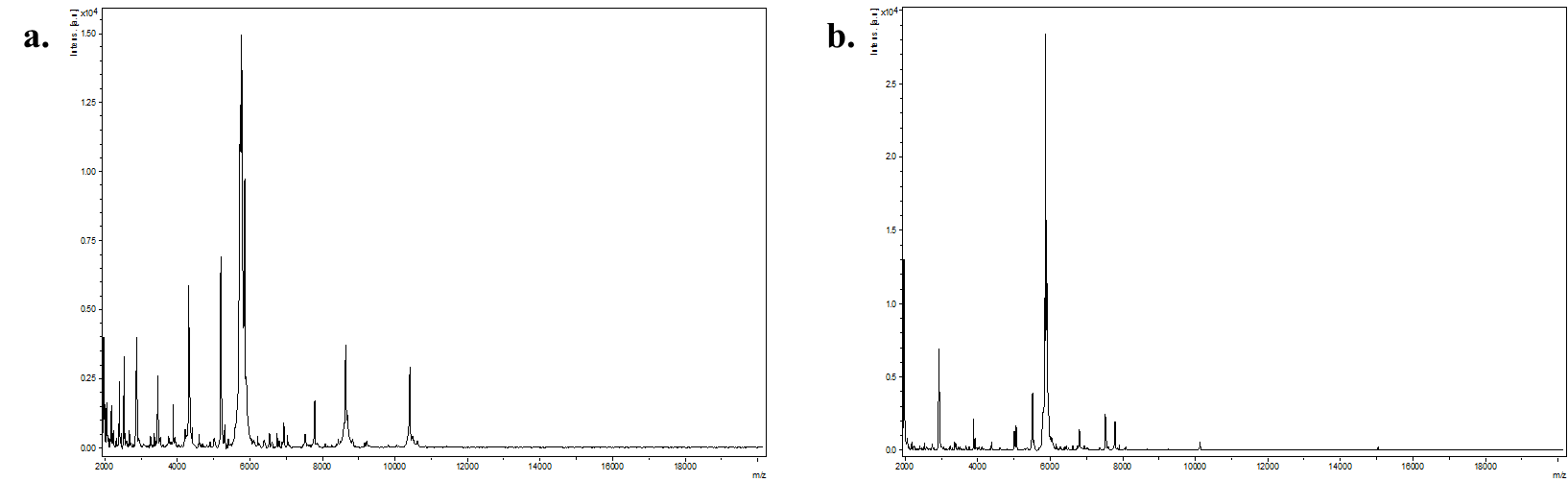
**

**Figure S1.** MALDI-TOF MS reference spectrum of (a) *Anaerococcus ihuae* sp. nov., strain Marseille-Q5893 and (b) ***Mediannikoviicoccus*** *vaginalis* gen. nov., sp. nov., strain Marseille-Q5883. The reference spectrum was generated by comparison of spectra from 6 individual colonies using the Biotyper 3.0 software.

**
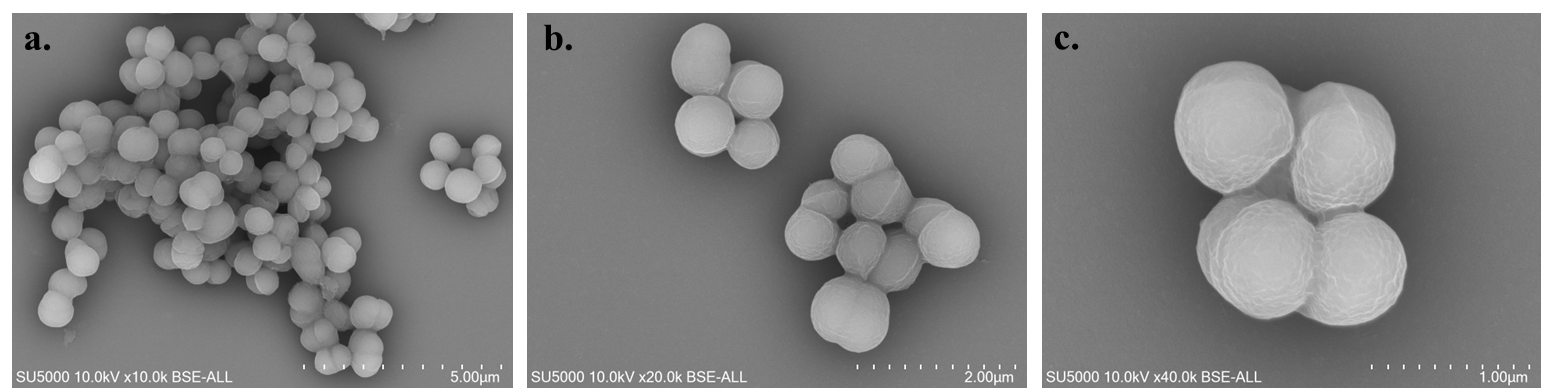
**

**Figure S2.** Scanning electron microscopy of *Anaerococcus ihuae* sp. nov., strain Marseille-Q5893 (a, b, and c).

**Tables**

**Table S1.** Characteristics of strains Marseille-Q5893 and Marseille-Q5883.

| Properties | Strain Marseille-Q5893 | Strain Marseille-Q5883 |
| --- | --- | --- |
| Genus name | *Anaerococcus* | ***Mediannikoviicoccus*** |
| Species name | *Anaerococcus ihuae* | ***Mediannikoviicoccus*** *vaginalis* |
| Status | sp. nov. | gen. nov., sp. nov. |
| Designation of the type strain | CSUR Q5893 | CSUR Q5883 |
| Strain collection numbers | CECT 30496 | CECT 30497 |
| 16S rRNA gene accession number | OM728648 | OM728652 |
| Genome accession number | CAKMRU010000001 | CAKMRI010000001 |
| Genome size | 1,831,371 bp | 1,997,945 bp |
| G + C (mol %) | 29.4 | 33.6 |
| Origin | Marseille, France | Marseille, France |
| Date of isolation | May 2021 | June 2021 |
| Source of isolation | Human vaginal sample | Human vaginal sample |
| Conditions used for standard cultivation | Columbia agar + with 5% sheep blood for 48 h of incubation | Columbia agar + with 5% sheep blood for 48 h of incubation |
| Gram stain | Positive | Positive |
| Cell shape | Cocci | Cocci |
| Cell size (diameter) | 0.75 μm | 0.63 μm |
| Motility | Non-motile | Non-motile |
| Sporulation | Non-sporulate | Non-sporulate |
| Colony morphology | Circular white opaque | Circular white translucent |
| Temperature optimum | 37°C | 37°C |
| pH range | 6-7.5 | 6-7.5 |
| O_2_ requirement | Strictly anaerobe | Facultative anaerobe |
| Oxidase | Negative | Negative |
| Catalase | Negative | Negative |
| Salinity range | 0-20% | 0-20% |

**Table S2.** API 50CH, API 20A, and API ZYM (bioMérieux) results for *Anaerococcus ihuae* sp. nov., strain Marseille-Q5893 and ***Mediannikoviicoccus*** *vaginalis* gen. nov., sp. nov., strain Marseille-Q5883.

|  | **Marseille-Q5893** | **Marseille-Q5883** |
| --- | --- | --- |
| **API ZYM** |  |  |
| Acid phosphatase | + | - |
| Naphthol-AS-BI-phosphohydrolase | - | + |
| Leucine arylamidase | + | + |

+, positive; -, negative.

Both strains had negative reactions with API ZYM strips for phosphatase alkaline, esterase (C4), esterase lipase (C8), lipase (C14), valine arylamidase, cystine arylamidase, trypsin, α-chymotrypsin, α-galactosidase, β-galactosidase, β-glucuronidase, α-glucosidase, β-glucosidase, N-acetyl-β-glucosaminidase, α-mannosidase, α-fucosidase.

Both strains do not produce acid, using API 50 CH strips from glycerol, erythritol, D-arabinose, L-arabinose, D-ribose, D-xylose, L-xylose, D-adonitol, methyl β-D-xylopyranoside, D-galactose, D-glucose, D-fructose, D-mannose, L-sorbose, L-rhamnose, dulcitol, inositol, D-mannitol, D-sorbitol, methyl α-D-mannopyranoside, methyl-α-D-glucopyranoside, N-acetylglucosamine, amygdaline, arbutine, esculin ferric citrate, salicine, D-cellobiose, D-maltose, D-lactose, D-melibiose, D-saccharose, D-trehalose, inulin, D-melezitose, D-raffinose, amidon, glycogen, xylitol, gentiobiose, D-turanose, D-xylose, D-tagalose, D-fucose, L-fucose, D-arabitol, L-arabitol, potassium gluconate, potassium 2-cetogluconate, potassium 5-cetogluconate.

Both strains had negative reactions with API 20A strips for L-tryptophane, urea, D-glucose, D-mannitol, D-lactose, D-saccharose, D-maltose, salicin, D-xylose, L-arabinose, gelatin, esculin ferric citrate, glycerol, D-cellobiose, D-mannose, D-melezitose, D-raffinose, D-sorbitol, L-rhamnose, D-trehalose.

**Table S3.** Cellular fatty acid composition (%) of *Anaerococcus ihuae* sp. nov., strain Marseille-Q5893 and ***Mediannikoviicoccus*** *vaginalis* gen. nov., sp. nov., strain Marseille-Q5883 compared with other of closely related species.

| **Fatty acids** | **Name** | **1** | **2** | **3** | **4** | **5** | **6** |
| --- | --- | --- | --- | --- | --- | --- | --- |
| C_16:0_ | Hexadecanoic acid | 61.0 | 52.7 | 59.2 | 62.3 | 33.0 | ֊ |
| C_18:1n9_ | 9-octadecenoic acid | 20.2 | 28.2 | 18.8 | 15.1 | ND | ֊ |
| C_14:0_ | Tetradecanoic acid | 5.8 | 2.1 | 6.9 | 8.2 | 4.4 | 2.4 |
| C_18:2n6_ | 9,12-octadecadienoic acid | 4.8 | 10.1 | 6.5 | 6.9 | ֊ | ֊ |
| C_18:0_ | Octadecanoic acid | 3.6 | 3.6 | 5.7 | 2.5 | 16.2 | 7.6 |
| C_10:0_ | Decanoic acid | 1.3 | ֊ | 1.2 | ֊ | ֊ | ֊ |
| C_15:0_ | Pentadecanoic acid | 1.3 | TR | 1.0 | ֊ | ֊ | ֊ |

These values represent the mean peak area percentage calculated from the analysis of FAMEs; TR = trace amounts < 1%.

Strains: **1**, *Anaerococcus ihuae* Marseille-Q5893; **2**, *Anaerococcus* *rubiinfantis* mt16 (Alou et al. 2016); **3**, *Anaerococcus vaginalis* CCUG 31349 (Alou et al. 2016); **4**, ***Mediannikoviicoccus*** *vaginalis* Marseille-Q5883; **5**, *Peptoniphilus duerdenii* WAL 18896 (Johnson et al. 2014); **6**, *Finegoldia magna* (Wells and Field 1976).
